# Changes in Metabolome and Nutritional Quality of *Lycium barbarum* Fruits from Three Typical Growing Areas of China as Revealed by Widely Targeted Metabolomics

**DOI:** 10.3390/metabo10020046

**Published:** 2020-01-26

**Authors:** Yajun Wang, Xiaojie Liang, Yuekun Li, Yunfang Fan, Yanlong Li, Youlong Cao, Wei An, Zhigang Shi, Jianhua Zhao, Sujuan Guo

**Affiliations:** 1 Key Laboratory for Silviculture and Conservation, Ministry of Education, Beijing Forestry University, Beijing 100083, China; yajun817@163.com; 2 National Wolfberry Engineering Research Center, Yinchuan 750002, China; liangxj303@163.com (X.L.); linda28772877@163.com (Y.L.); majoriefyf@163.com (Y.F.); lylsxfp@163.com (Y.L.); youlongchk@163.com (Y.C.); angouqi@163.com (W.A.); zhaojianhua0943@163.com (J.Z.); 3 Ningxia Academy of Agriculture and Forestry Sciences, Yinchuan 750002, China; shizhigang76@163.com

**Keywords:** wolfberry, metabolic profiling, ecological factors, bioactive compounds, health benefits

## Abstract

This study aimed at assessing the climatic factors influencing the wolfberry fruit morphology, and the composition of its nutritious metabolites. The cultivar Ningqi1, widely grown in Northwest China was collected from three typical ecological growing counties with contrasting climatic conditions: Ningxia Zhongning (NF), Xinjiang Jinghe (XF) and Qinghai Nomuhong (QF). During the ripening period, 45 fruits from different plantations at each location were sampled. A total of 393 metabolites were detected in all samples through the widely targeted metabolomics approach and grouped into 19 known classes. Fruits from QF were the biggest followed by those from XF and NF. The altitude, relative humidity and light intensity had negative and strong correlations with most of the metabolites, suggesting that growing wolfberry in very high altitudes and under high light intensity is detrimental for the fruit nutritional quality. Soil moisture content is highly and negatively correlated with vitamins, organic acids and carbohydrates while moderately and positively correlated with other classes of metabolites. In contrast, air and soil temperatures exhibited positive correlation with majority of the metabolites. Overall, our results suggest high soil and air temperatures, low altitude and light intensity and moderate soil moisture, as the suitable conditions to produce *Lycium* fruits with high content of nutritious metabolites.

## 1. Introduction

*Lycium barbarum* L. known as goji berry or wolfberry is a tree species belonging to the Solanaceae family. It is widely cultivated in the arid and semi-arid areas of North and South America, Africa and Eurasia [1]. In China, there are seven species and three varieties that are mainly distributed in the northwest and north parts of the country [2]. In 2008, about 101,171 ha were grown in China with 7845 kg ha^-1^ as the maximum of yield from elite genotypes [3] while in 2019 the wolfberry cultivated area is over more than 1.33 10^5^ ha [4]. Wolfberry is much appreciated for its nutritional, cosmetic and pharmaceutic applications [5,6]. In China, the fruit is consumed raw, cooked or dried. It is mainly sun-dried and used in the common dishes like soups, hot pots, herbal teas, wines and medicines [7]. 

The fruit compounds such as vitamins, minerals and antioxidants are known to fight against the free radicals and inflammation, and boost the immune system [6,8]. A wolfberry can provide twice the daily recommended dose of vitamins A and C [9]. Interestingly, wolfberry is specifically enriched with antioxidants called LBP (*Lycium barbarum* polysaccharides), the key bioactive elements in the fruit providing various impressive health benefits [10,11]. LBP are reported to exhibit antitumor activity (anticancer) and to be effective in the protection against diabetes and hyperglycemia [12,13,14]. In addition, wolfberry fruit provide many amino acids mainly about 11 essential amino acids more than other common berries [9,15]. Moreover, many other compounds were isolated from wolfberry plant including lignanamides, alkaloids, flavonoids, cyclic peptides, lignans, terpenes and phenolic glycosides [2,16].

Yajun et al. [4] reported that the wolfberry cultivation areas in China are characterized by very different climatic conditions, differing in particular on the altitude, drought, salinity, sunshine hours, diurnal temperature difference and soil quality that are able to affect the growth of the plant and the metabolites from photosynthesis. Dong et al. [17] analyzed the total flavonoids content in the leaf from the cultivated and wild *L. barbarum.* The wild types were collected from four locations in the Northwest region of China. These authors observed higher total flavonoids content in the cultivated than wild types and within the wild types the performance differed according to their origin of provenance. Zheng et al. [18] have also shown that *Lycium barbarum* and *Lycium chinense* cultivars and their origin of provenance, as well as their microenvironmental conditions (the levels of soil salt, pH, organic matter and available nitrogen) influenced the sugar composition of the wolfberry fruit in China. Moreover, Mocan et al. [19] reported that polyphenols compounds in the wolfberry fruits from European cultivars, were strictly dependent on the geographic origin (soil type and climate), the type of cultivar, harvesting period and homogenization treatment applied during the chemical analysis. In Turkey, Çolak et al. [20] reported that the pomological, biochemical and phytochemical properties of the wolfberry fruit were affected by the harvest time and location of cultivation. Many studies have also shown the environment factors influence on the phytochemical constituents of other tree species or vegetables crops [21,22,23,24,25].

However, in the previous researches performed in China, very few focused on a large sample size and assessed the influence of typical ecological growing areas contrasting in their climatic conditions, on a large number of metabolites detected. Therefore, the present study aimed at assessing the climatic factors influencing the wolfberry fruit morphology, and the composition and quality of its bioactive components through the widely targeted metabolomics approach. The cultivar Ningqi1, widely grown in the wolfberry growing areas in Northwest China was collected from three typical ecological growing areas and a large number of metabolites (393) were detected using the widely targeted metabolomics approach. The findings will inform on the nutritional and medicinal qualities of wolfberry fruit from different cultivated areas in China.

## 2. Results

### 2.1. Meteorological Conditions of the Growing Areas and Effects on Wolfberry Fruit Dimensions

**Figure 1:**
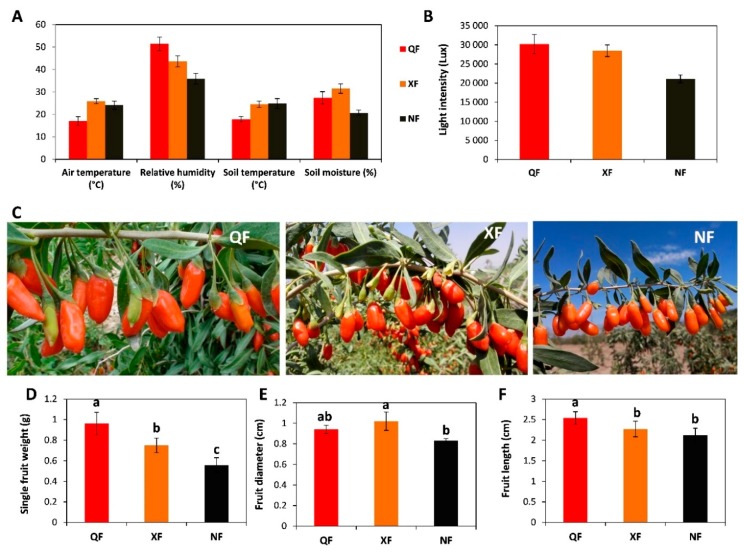
Meteorological conditions of three typical growing areas of wolfberry in China and measurement of the size of fruits obtained from each location. (**A**) Average air temperature, relative humidity, soil temperature and soil moisture content in the three locations during the fruit growth period; (**B**) light intensity in the three locations during the fruit growth period; (**C**) pictures of the wolfberry fruits at each location during ripening period; (**D**) single fruit weight, (**E**) fruit diameter and (**F**) fruit length. QF, NF and XF represent Qinghai, Ningxia and Xinjiang growing area, respectively. Bars represent the average values with SD from various measurements. The letter above bars represent the results of statistical tests and bars with different letters are significantly different at *p* <0.05.

Wolfberry fruits grown in three typical wolfberry growing areas, including Qinghai Nomuhong County (QF), Ningxia Zhongning County (NF) and Xinjiang Jinghe County (XF) [4], were investigated in this study. The altitudes of QF, NF and XF were 2783 m, 1123 m and 290 m, respectively. Various meteorological parameters were collected during wolfberry fruit growth from June to August 2018 at the three planting areas and data are summarized in Figure 1A. The air and soil temperatures of QF were lower than those of NF and XF. QF had also very high relative air humidity and soil moisture content. XF was characterized by high air and soil temperatures, relative air humidity and moderate soil moisture content. NF also had high air and soil temperatures but had low relative air humidity and soil moisture content. The light intensity was also recorded in the three regions and as shown in Figure 1B, QF had also the highest light intensity, followed by XF and finally NF. Overall, it could be observed that climatic conditions from these three wolfberry planting ecological zones were clearly different.

Differences in size of fruits from the three producing areas were also investigated during the ripening period. Overall, fruits from QF were the biggest followed by those from XF and the smallest fruits were obtained in NF (Figure 1C–F). These results proved that climatic conditions had significant effects on wolfberry fruit size.

### 2.2. Overview of the Metabolic Profiles of Wolfberry Fruits Collected from Three Different Planting Areas

In order to evaluate the effect of the different climatic conditions on wolfberry fruit metabolome, the fruit samples collected from the three planting areas namely NF, QF and XF, were used for metabolic profiling based on the widely targeted metabolomics approach. Totally, 393 metabolites were detected in all samples and grouped into 19 known classes based on the structure of the metabolites (Table 1, Table S1). Wolfberry fruit is rich in metabolites belonging to the classes of polyphenols (flavones, flavoves, flavonols, flavonoids, phenolamides and phenylpropanoids), organic acids and derivatives, amino acid and derivatives and lipids.

**Figure 2:**
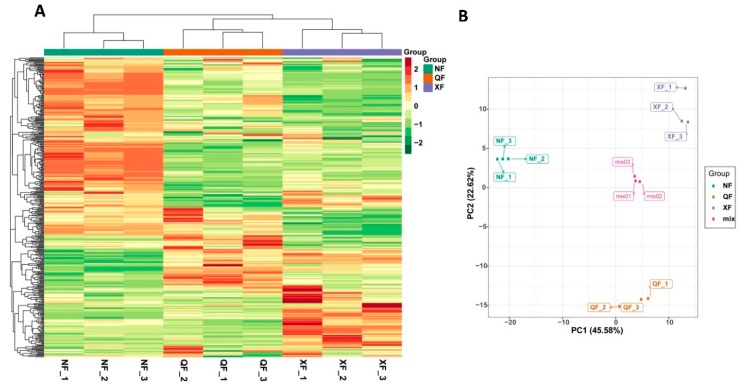
Overview of global metabolic profiles in wolfberry fruits collected from three planting areas. (**A**) Heatmap hierarchical clustering and (**B**) principal component (PC) analysis. NF, XF and QF represent Ningxia, Xinjiang and Qinghai planting areas, respectively. Mix represents the mixed samples used for quality control. Data shown are the log_2_ fold change of the metabolite ion intensity.

The metabolite ion intensity data was used to construct a hierarchical clustering heatmap and also to perform principal component analysis (PCA) of the samples. The hierarchical heatmap showed that all biological replicates from the same planting area were clustered together, denoting the reliability of the metabolic profiling data (Figure 2A). Moreover, the samples from the three planting areas were clearly separated, which implies that a large number of metabolites are differentially accumulated between these samples. In Figure 2B, the PCA result indicates that the mixed samples used for quality control were all near to the origin (0:0), suggesting a very little technical variability between runs. In addition, we observed a clear separation between the different samples, with the first two PC explaining over 68% of the total variability. Samples from NF and XF were distinguished by PC1 while samples from QF were separated from those from the two other planting areas by PC2.

### 2.3. Differentially Accumulated Metabolites between Fruits from the Three Locations

**Figure 3:**
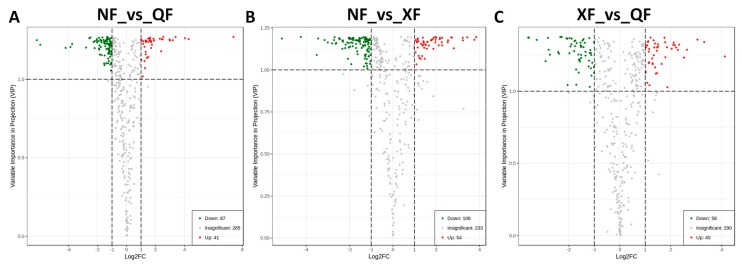
Volcano plots depicting the up- and down- accumulated metabolites between pairs of samples from different growing regions. (**A**) between NF and QF; (**B**) between NF and XF; (**C**) between XF and QF. Compounds with variable importance in projection (VIP) ≥ 1 and fold change ≥2 or fold change ≤ 0.5 were declared significant. QF, NF and XF represent Qinghai, Ningxia and Xinjiang locations, respectively.

To detect wolfberry fruit metabolites that were significantly changed according to the climatic conditions from the three planting areas, we compared each metabolite ion intensity between samples from different growing areas and the compounds with variable importance in projection (VIP) ≥1 and fold change ≥2 or fold change ≤0.5 [26] were retained as differentially accumulated metabolites (DAM). This analysis resulted into 128 DAMs for NF_vs_QF, 160 DAMs for NF_vs_XF and 103 DAMs for XF_vs_QF (Figure 3A–C). Overall, the contents of approximately 2/3 of the global metabolome (232 metabolites) were significantly altered (Table S2), showing that the growing environments highly influenced the metabolite content in wolfberry fruit.

**Figure 4:**
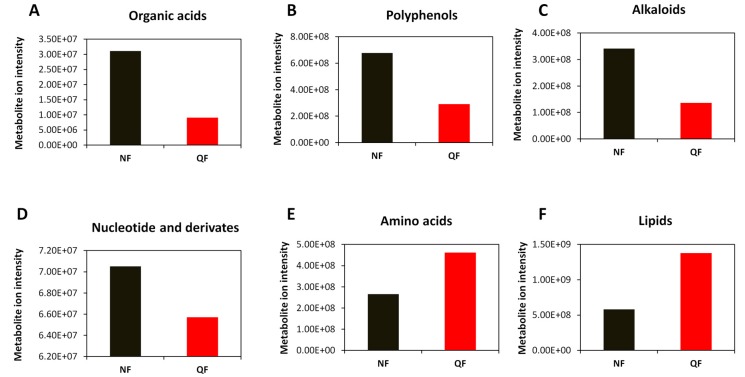
Comparison of the total ion intensity of various classes of metabolites between fruits from Ningxia (NF) and Qinghai (QF). (**A**) Organic acids, (**B**) polyphenols, (**C**) alkaloids, (**D**) nucleotides and derivatives, (**E**) amino acids and (**F**) lipids. Bars represent the sum of ion intensity of all metabolites belonging to each class.

**Figure 5:**
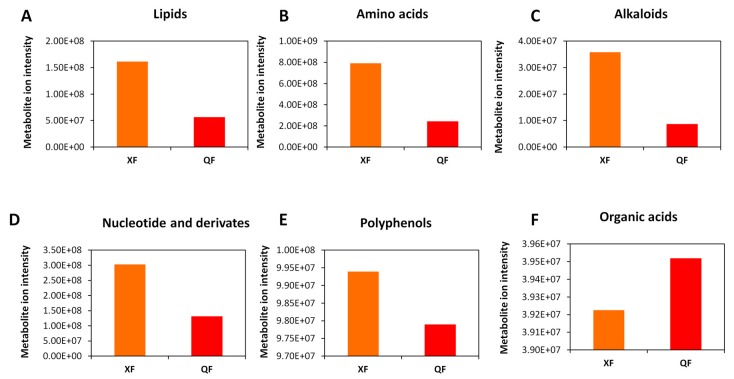
Comparison of the total ion intensity of various classes of metabolites between fruits from Xinjiang (XF) and Qinghai (QF). (**A**) Lipids, (**B**) amino acids, (**C**) alkaloids, (**D**) nucleotides and derivatives, (**E**) polyphenols and (**F**) organic acids. Bars represent the sum of ion intensity of all metabolites belonging to each class.

**Figure 6:**
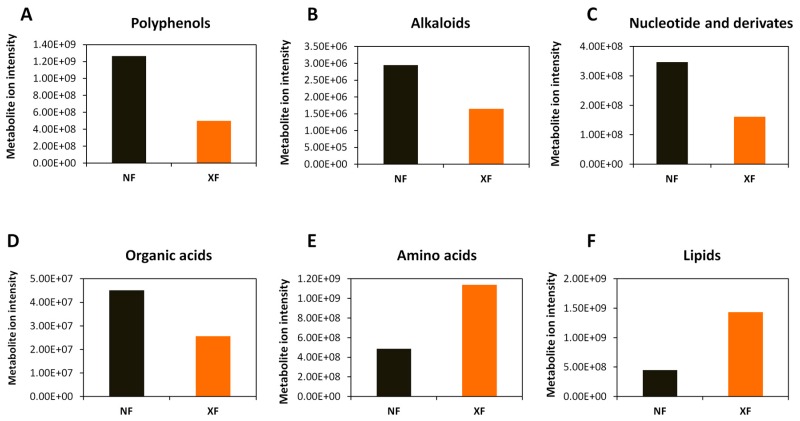
Comparison of the total ion intensity of various classes of metabolites between fruits from Ningxia (NF) and Xinjiang (XF). (**A**) Polyphenols, (**B**) alkaloids, (**C**) nucleotides and derivatives, (**D**) organic acids, (**E**) amino acids and (**F**) lipids. Bars represent the sum of ion intensity of all metabolites belonging to each class.

We observed a significant decrease of metabolite ion intensity in fruits from QF as compared to NF. Globally, alkaloids, nucleotide and derivates, polyphenols (flavones, flavoves, flavonols, flavonoids, phenolamides and phenylpropanoids) and organic acids were more enriched in wolfberry fruits from NF while lipids and amino acids were highly concentrated in fruits from QF (Figure 4). Similarly, by comparing the metabolite ion intensity between fruits from XF and QF, we noticed a strong decrease in the content of alkaloids, amino acids, lipids and nucleotide and derivates in fruits from QF compared to those from XF. The polyphenol content was slightly decreased in QF while the organic acids were slightly increased in fruits from QF (Figure 5). Although the highest number of DAMs was observed between wolfberry fruit samples from NF and XF, the amplitude of changes of metabolite ion intensity was not very significant. We found that fruits from NF contained two times polyphenols, alkaloids, amino acids and organic acids than fruits from XF. In contrast, XF wolfberry fruits were enriched in lipids and amino acids (Figure 6).

**Figure 7:**
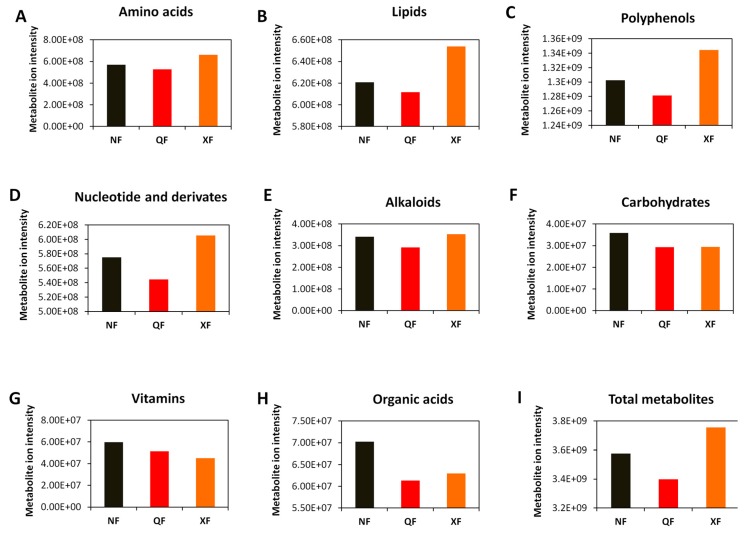
Comparison of the total ion intensity of various classes of metabolites between fruits from Ningxia (NF), Qinghai (QF) and Xinjiang (XF). (**A**) Amino acids, (**B**) lipids, (**C**) polyphenols, (**D**) nucleotides and derivatives, (**E**) alkaloids, (**F**) carbohydrates, (**G**) vitamins, (**H**) organic acids and (**I**) total metabolites. Bars represent the sum of ion intensity of all metabolites belonging to each class.

To better understand the changes in wolfberry fruit metabolite ion intensity across the three planting areas, we grouped the 232 DAMs (Table S2) into major classes (eight in total) and compared them. As shown in Figure 7, wolfberry fruits from XF were the richest in polyphenols, amino acids, lipids, alkaloids, nucleotide and derivates. Wolfberry fruits from NF were rich in carbohydrates, organic acids and vitamins (Figure 7). Finally, the fruits from QF seem to be less nutritious compared to samples from XF and NF as they have the lowest metabolite ion intensity (Figure 7).

By cross-comparing the different DAMs detected between samples from the three growing areas, only 13 metabolites were constitutively changed across of all the locations, indicating that these metabolites are highly sensitive to climatic fluctuations and may be used as biomarkers to rapidly identify the origins (planting areas) of wolfberry fruits in the market (Figure S1, Table 2).

### 2.4. Correlations between Classes of Metabolites and Environmental Factors

We further examined the relationships between the wolfberry metabolites and the meteorological factors of the three production areas using the bivariate correlation analysis (Table 3). The altitude, relative air humidity and light intensity had negative and strong correlations with most of the metabolites, suggesting that growing wolfberry in very high altitude and under high light intensity is detrimental for the fruit nutritional quality. Soil moisture content is highly and negatively correlated with vitamins, organic acids and carbohydrates while moderately and positively correlated with other classes of metabolites. In contrast, the air and soil temperatures exhibited positive correlation with majority of the metabolites. Altogether, our data suggest that high soil and air temperatures, low altitude and light intensity and moderate soil moisture are the favorable climatic conditions for obtaining wolfberry fruits with high ion intensity of important and nutritious metabolites.

## 3. Discussion

In this study, the cultivar Ningqi1, widely grown in the wolfberry growing areas in Northwest China was collected from three typical ecological growing areas with contrasting climatic conditions. The fruits morphology and its nutritional and bioactive compounds were assessed. The results are discussed with regard to the ecological growth conditions enabling to produce fruit with a high content of nutritional and health-promoting metabolites.

Many studies reported the Northwest region of China as the most wolfberry growing area of the country [27,28]. Particularly, Ningxia Province is mentioned as recorded in 2015 the largest production, equivalent to 45% of the national wolfberry production and having the premium quality [29]. This location in addition to Xinjiang Jinghe (XF) and Qinghai Nomuhong Counties (QF) were specifically reported in the previous studies to have contrasted climatic conditions [4,30]. This was also confirmed in the present study through the climatic parameters recorded in 2018, mainly altitude, air and soil temperatures, relative air humidity and soil moisture content. 

The fruit morphological traits recorded including the single fruit weight, diameter and length, showed differences in size of fruits from the three producing areas. Overall, fruits from QF were the biggest followed by those from XF and the smallest fruits were obtained in NF. Yao et al. [30] assessing the fruit morphology from four climatic regions in China including our studied locations, reported a significant difference in fruit size, weight and redness. They also observed the largest and heaviest fruit from the Plateau climatic region (Qinghai) followed by the arid (Xinjiang) and semi-arid region (Ningxia), and explained this higher performance of the Plateau region by the drastic fluctuation of the temperature between day and night in this region. Wetters et al. [31] studying the diversity of wolfberry accessions from various regions in the world, showed significant a difference in fruit size. Our results confirm the significant effect of the typical growing area on the wolfberry fruit morphology. The fruit shape is often used by the stakeholders to determine its origin of cultivation and its price in the market and the larger fruits are more expensive [30].

The widely targeted metabolomics approach was successfully used to detect in all samples, 393 metabolites, which were grouped into 19 known classes. Wolfberry fruit is rich in metabolites belonging to the classes of polyphenols (flavones, flavoves, flavonols, flavonoids, phenolamides and phenylpropanoids), organic acids and derivatives, amino acid and derivatives and lipids. Similar groups of metabolites were obtained in the previous studies in *Lycium* fruit extracts [4,19,30,32]. Fruits collected from NF were more endowed in alkaloids, nucleotide and derivates, polyphenols and organic acids while lipids and amino acids were more concentrated in those from QF. Fruits from this latter location, were poorer in alkaloids, amino acids, lipids and nucleotide and derivates than those from XF. The polyphenols content was slightly decreased in QF while the organic acids were slightly increased in fruits from QF. In short, most of the metabolites detected, differentiated the growing areas where the wolfberry fruits were sampled. Yossa Nzeuwa et al. [28] comparing the metabolic profiling of *Lycium* dry fruit extracts from the main growing areas in China and Nepal, showed a slight difference in contents of metabolites between the growing areas. Bondia-Pons et al. [33] performed the metabolomic profiling to discriminate samples from four different geographic origins using the fruit phytochemical contents, were able to successfully distinguish Inland Chinese, Inner Mongolian and Tibetan wolfberry fruits extracts. Moreover, on the wolfberry fruits from European cultivars, Mocan et al. [19] reported that polyphenols compounds were strictly dependent on the geographic origin (soil type and climate), the type of cultivar, harvesting period and homogenization treatment applied during the chemical analysis. Several studies highlighted goji berry fruits cultivated in different countries like Italy, Serbia and Greece, as endowed, in different proportions, with important fatty acids, phytosterols, carotenoids and phenolic compounds that can be exploited for nutritional and pharmaceutical purposes [34,35,36,37,38]. The ecological factors influence on the metabolite constituents was also mentioned for other tree species. Bokulich et al. [39] distinguished viticultural areas and individuals vineyards within Napa and Sonoma Counties in California, USA, using the grape microbiota and wine metabolites profiles. Similarly, da Silva Taveira et al. [40] differentiated coffee genotypes from different origins using metabolic profiling. However, Yao et al. [30] performing the metabolomic profiling, were able to discriminate two *Lycium* species (*L. barbarum* and *L. chinense*) but not the fruits samples collected from different cultivation areas. In short, the metabolomic profiling performed in the present study, was able to distinguish Ningxia, Qinghai Nomuhong and Xinjiang Jinghe counties where the fruit sampling was done. These counties were mainly contrasted for their altitude, air and soil temperatures, relative air humidity and soil moisture content. The altitude, relative air humidity and light intensity had negative and strong correlations with most of the metabolites, suggesting that growing a wolfberry in a very high altitude and under high light intensity is detrimental for the fruit nutritional quality. Negative influence of altitude gradient was also shown on the growth and synthesis of some metabolites (photosynthetic pigments, malondialdehyde, etc.) in *Leymus secalinus*, assessed across eight different altitudes in the Qinghai–Tibetan Plateau [41]. However, these authors observed that proline, soluble sugar contents and other markers of stress increased with elevation. This suggests an osmotic adjustment mechanism triggered by the plants in the alpine regions to adapt and survive to such conditions. Moreover, high altitude and the corresponding lower temperatures were also shown to affect the functional leaf number and the yield of plantains in North Kivu, DR Congo [42]. A high altitude effect observed in the present study on the growth and some metabolites accumulation in a wolfberry, could be overcome by searching in a large diversity, genotypes with ability to adapt to high altitude and lower temperatures and use them to improve the cultivars grown in high altitude areas. Moreover, soil moisture content was found to be highly and negatively correlated with vitamins, organic acids and carbohydrates while moderately and positively correlated with other classes of metabolites. High soil moisture content seems to dilute the vitamins, organic acids and carbohydrates content in the wolfberry plants. Therefore, from all above, high soil and air temperatures, low altitude and light intensity and moderate soil moisture are the favorable climatic conditions for producing wolfberry fruits with high content of important and nutritious metabolites. Besides these conditions, the nature of the soil was also emphasized [43]. According to these authors, slightly alkaline soils are more favorable for growing a wolfberry than acidic soils. The plant is able to grow on a wide range of soil types but a light loam soil is the most preferable and waterlogging is to be avoided.

## 4. Materials and Methods

### 4.1. Plant Material and Sampling Areas

In the present study, *Lycium barbarum* cv. Ningqi1 was used as the experimental material. This cultivar is widely grown in the wolfberry growing areas in Northwest China [44]. The fruit samples were collected from three typical ecological growing areas with contrasting climatic conditions: Ningxia Zhongning County (NF), Xinjiang Jinghe County (XF) and Qinghai Nomuhong County (QF). To collect meteorological data, TT-QXZ weather stations were installed at each location and automatically recorded data on air temperature, air relative humidity, light intensity, soil relative humidity and soil temperature during the fruit growth period in 2018. At each location, three plantations were randomly selected. Field management procedures at each location were in accordance with the conventional way. The sample collection was conducted from June to August 2018 based on eight-year-old Ningqi1 trees, during the peak harvest time. Approximately, 50 fruits from five trees in each plantation were mixed to represent one biological replicate. Samples from the three plantations represent therefore three biological replicates for each location (NF, XF or QF). In total, nine samples were collected, frozen immediately in liquid nitrogen in the field, transported to the laboratory and then stored at −80 °C until further use.

### 4.2. Analysis of the Fruit Dimensions

During the ripening period, 45 fruits from different plantations at each location were harvested and used to measure single fruit weight (g), diameter (cm) and length (cm) with a Vernier caliper (±0.1 mm). Analysis of variance to test the significant difference of fruit dimensions between the three locations was computed in the R software version 3.3.2.

### 4.3. Metabolic Profiling

The sample preparation, extract analysis, metabolite identification and quantification were performed at Wuhan MetWare Biotechnology Co., Ltd, Wuhan, China, following their standard procedures and previously fully described by Yuan et al. [44].

### 4.4. Extraction of Samples’ Metabolites

The frozen samples were crushed using a mixer mill (MM 400, Retsch) with a zirconia bead for 1.5 min at 30 Hz. Then, the sample powder (100 mg) was extracted at 4 °C with 1 mL 70% aqueous methanol. Following centrifugation at 10,000 × *g* for 10 min, the extracts were absorbed (CNWBOND Carbon-GCB SPE Cartridge, 250 mg, 3 mL; ANPEL, Shanghai, China) and filtrated (SCAA-104, 0.22 μm pore size; ANPEL, Shanghai, China) before LC-MS analysis.

### 4.5. Metabolite Identification and Quantification

The sample extracts were analyzed using an LC-ESI-MS/MS system (HPLC, Shim-pack UFLC SHIMADZU CBM30A system, Kyoto, Japan; MS, Applied Biosystems 6500 Q TRAP, San Diego, CA, USA). The HPLC effluent was alternatively connected to an electrospray ionization (ESI)-triple quadrupole-linear ion trap–MS/MS system (Applied Biosystems 4500 Q TRAP, San Diego, CA, USA). The analytical conditions were following descriptions of Chen et al. [40].

Metabolite identification was based on the Metware MWDB database, following their standard metabolic operating procedures. Metabolite quantification was carried out using multiple-reaction monitoring (MRM) [40].

### 4.6. Metabolite Data Analysis

Before the data analysis, quality control (QC) analysis was conducted to confirm the reliability of the data. The QC sample was prepared by the mixture of sample extracts and inserted into every three samples to monitor the changes in repeated analyses. Data matrices with the intensity of the metabolite features from the nine samples were uploaded to the Analyst 1.6.1 software (AB SCIEX, Ontario, Canada) for statistical analyses. The supervised multivariate method, partial least squares-discriminant analysis (PLS-DA), was used to maximize the metabolome differences between the pair of samples. The relative importance of each metabolite to the PLS-DA model was checked using the parameter called variable importance in projection (VIP). Metabolites with VIP ≥1 and fold change ≥2 or fold change ≤0.5 were considered as differential metabolites for group discrimination [44]. Pearson correlation analysis, principal component analysis (PCA) and Ward’s hierarchical clustering heatmap were performed in the R software version 3.3.2 (www.r-project.org). The PCA was based on three components and the correlation matrix.

## 5. Conclusions

The present study assessed the climatic factors influencing the wolfberry fruit morphology, and the composition and quality of its bioactive constituents. The cultivar Ningqi1, widely grown in the wolfberry growing areas in Northwest China was collected from three typical ecological growing areas with contrasting climatic conditions. The widely targeted metabolomics approach performed, detected 393 metabolites in all samples and grouped into 19 known classes. The fruit is endowed in metabolites belonging to the classes of polyphenols, organic acids and derivatives, amino acid and derivatives and lipids. Significant influence of ecological factors was observed on the fruit morphology and its bioactive compounds. The altitude, relative air humidity and light intensity had negative and strong correlations with most of the metabolites. High soil moisture content highly and negatively influenced the vitamins, organic acids and carbohydrates content of wolfberry fruit. On contrary, high air and soil temperatures were favorable for the synthesis of the majority of metabolites. In conclusion, high soil and air temperatures, low altitude and light intensity and moderate soil moisture are the favorable climatic conditions for producing wolfberry fruits with high content of nutritious metabolites. This was a preliminary study since the samples collection was limited to a one-year growth period. Further studies are needed to confirm the trend observed by taking into account environmental conditions affecting the phytochemical composition of the crop such as pedo-climatic conditions and farming practices. Further studies are also needed to improve the existing cultivars to cope with the harsh conditions prevailing in high altitude growing areas.

## Figures and Tables

**Table 1:** Classification of the detected metabolites in wolfberry fruit into major classes.

Class	Number of Compounds	Class	Number of Compounds
Organic acids and derivatives	60	Vitamins and derivatives	13
Amino acid and derivatives	53	Flavanone	11
Lipids	52	Carbohydrates	10
Flavone	37	Alcohols	10
Nucleotide and derivates	37	Anthocyanins	7
Phenylpropanoids	26	Indole derivatives	3
Flavonol	17	Polyphenol	3
Alkaloids	17	Flavonoid	3
Phenolamides	17	Sterides	3
Others	13	Terpene	1

**Table 2:** List of metabolites showing constitutive differential accumulation between wolfberry fruits from three planting areas.

Compounds	Class	NF	QF	XF	Log2 FC (NF_vs_XF)	Log2 FC (NF_vs_QF)	Log2 FC (XF_vs_QF)
LysoPC 16:2 (2n isomer)	Lipids	9.75 × 10^5^	2.88 × 10^6^	1.40 × 10^7^	3.84	1.56	−2.28
Phellodensin F	Others	1.69 × 10^5^	5.94 × 10^4^	1.90 × 10^4^	−3.15	−1.51	1.64
Protocatechuic acid O-glucoside	Polyphenol	1.34 × 10^6^	2.16 × 10^4^	7.04 × 10^4^	−4.25	−5.96	−1.71
1-O-beta-D-Glucopyranosyl sinapate	Phenylpropanoids	8.44 × 10^5^	3.76 × 10^5^	1.70 × 10^5^	−2.31	−1.17	1.14
Tricin O-saccharic acid	Flavone	2.71 × 10^5^	1.24 × 10^5^	4.92 × 10^4^	−2.46	−1.13	1.34
Tricin 7-O-hexoside	Flavone	2.95 × 10^5^	4.03 × 10^3^	2.70 × 10^4^	−3.45	−6.19	−2.74
2-Isopropylmalate	Organic acids and derivatives	8.72 × 10^5^	3.57 × 10^5^	4.22 × 10^6^	2.28	−1.29	−3.56
2’-Deoxyinosine-5’-monophosphate	Nucleotide and derivates	7.16 × 10^5^	7.50 × 10^5^	1.75 × 10^5^	1.29	3.39	2.10
Rosmarinic acid	Organic acids and derivatives	9.08 × 10^5^	3.87 × 10^4^	1.40 × 10^4^	−2.69	−1.23	1.46
(3,4-Dimethoxyphenyl) acetic acid	Organic acids and derivatives	3.99 × 10^5^	2.16 × 10^3^	1.63 × 10^4^	−1.29	−4.21	−2.91
Naringenin	Flavanone	1.47 × 10^5^	6.48 × 10^6^	2.69 × 10^6^	−2.45	−1.18	1.27
L-Pipecolic acid	Amino acid and derivatives	4.52 × 10^5^	4.13 × 10^5^	1.96 × 10^5^	2.11	3.19	1.08
Eriodictyol	Flavanone	2.43 × 10^5^	4.96 × 10^5^	5.99 × 10^4^	−2.02	1.03	3.05

**Table 3:** Correlation analysis between meteorological factors and major metabolite classes in wolfberry fruits.

Title	Alkaloids	Amino Acids	Carbohydrates	Polyphenols	Lipids	Nucleotides	Organic Acids	Vitamins
Altitude	**−0.99**	**−0.92**	−0.21	**−0.93**	**−0.88**	**−0.98**	−0.35	0.26
Air temperature	**1.00**	**0.86**	0.35	**0.87**	**0.80**	**0.95**	0.48	−0.12
Relative air humidity	−0.77	−0.32	**−0.88**	−0.33	−0.21	−0.51	**−0.94**	−0.56
Soil temperature	**0.98**	0.72	0.56	0.73	0.64	**0.85**	0.67	0.11
Soil moisture	0.04	0.56	**−0.92**	0.54	0.65	0.37	**−0.85**	**−1.00**
Light intensity	−0.51	0.02	**−0.99**	0.01	0.13	−0.18	**−1.00**	**−0.80**

Correlation values higher than 0.8 were marked in bold.

## Supplementary Materials

The following are available online at https://www.mdpi.com/2218-1989/10/2/46/s1. **Figure S1**. Venn diagram depicting the shared and unique differentially accumulated metabolites between pairs of wolfberry fruits samples from different planting areas. QF, NF and XF represent Qinghai, Ningxia and Xinjiang locations, respectively. Table S1. List of the metabolites detected in wolfberry fruits from different locations using the widely targeted metabolomics approach. QF, NF and XF represent Qinghai, Ningxia and Xinjiang locations, respectively. Mix represents the mixed samples used for quality control. Data represent the metabolite ion intensity. Table S2. List of the metabolites differentially accumulated between pairs of wolfberry fruit samples from different planting areas. QF, NF and XF represent Qinghai, Ningxia and Xinjiang locations, respectively. Data represent the metabolite ion intensity. TRUE means significantly different while FALSE represents no significant difference between pairs of samples.
