# Supplementary material for: Changes in Metabolome and Nutritional Quality of Lycium barbarum Fruits from Three Typical Growing Areas of China as Revealed by Widely Targeted Metabolomics
Source: Metabolites. 2020 Jan 26;10(2):46. doi: 10.3390/metabo10020046 (PMC7073637; doi:10.3390/metabo10020046)
Supplement: Supplementary file 1 [file metabolites-10-00046-s001.zip › Supplementary files.docx]

**Supplementary files.**


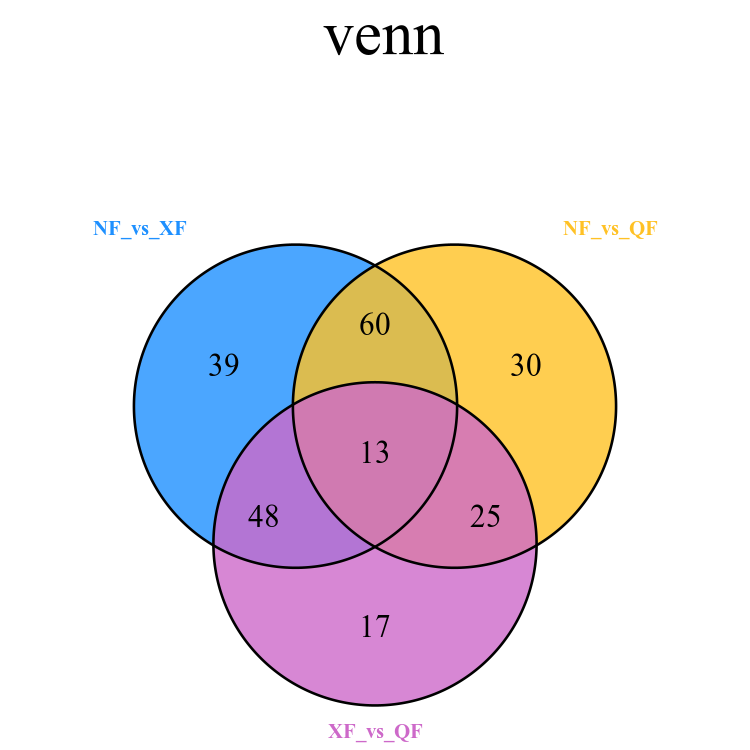


**Figure S1**. Venn diagram depicting the shared and unique differentially accumulated metabolites between pairs of wolfberry fruits samples from different planting areas. QF, NF and XF represent Qinghai, Ningxia and Xinjiang locations, respectively.

**Table S1**. List of the metabolites detected in wolfberry fruits from different locations using the widely-targeted metabolomics approach. QF, NF and XF represent Qinghai, Ningxia and Xinjiang locations, respectively. Mix represents the mixed samples used for quality control. Data represent the metabolite ion intensity.

**Table S2**. List of the metabolites differentially accumulated between pairs of wolfberry fruit samples from different planting areas. QF, NF and XF represent Qinghai, Ningxia and Xinjiang locations, respectively. Data represent the metabolite ion intensity. TRUE means significantly different while FALSE represents no significant difference between pairs of samples.
